# Cytotoxicity and radiosensitizing potency of Moscatilin in cancer cells at low radiation doses of X-ray and UV-C

**DOI:** 10.1007/s13205-021-02827-3

**Published:** 2021-05-20

**Authors:** Ipsita Pujari, Abitha Thomas, Jinsu Thomas, Niharika Jhawar, Kanive Parashiva Guruprasad, Padmalatha S. Rai, Kapaettu Satyamoorthy, Vidhu Sankar Babu

**Affiliations:** 1 Department of Plant Sciences, Manipal School of Life Sciences (MSLS), Manipal Academy of Higher Education (MAHE), Manipal, Udupi, Karnataka 576104 India; 2 Department of Ageing Research, Manipal School of Life Sciences, Manipal Academy of Higher Education, Manipal, Karnataka India; 3 Department of Biotechnology, Manipal School of Life Sciences, Manipal Academy of Higher Education, Manipal, Karnataka India; 4 Department of Cell and Molecular Biology, Manipal School of Life Sciences, Manipal Academy of Higher Education, Manipal, Karnataka India

**Keywords:** Apoptosis, Crude extract, Cytotoxicity, *Dendrobium ovatum*, Moscatilin, Radiosensitization

## Abstract

Moscatilin (stilbenoid) is a plant-derived anticancer compound, and it has mostly been isolated from threatened wild *Dendrobium* species. The present study attempts to evaluate the cytotoxicity of Moscatilin on several cancer cell lines through MTT assay. Additionally, it also aims towards estimating and comparing the radiosensitivity, cell-cycle progression, and apoptotic/necrotic effect induced by Moscatilin on different cell lines. The effects of Moscatilin was compared with another significant stilbenoid anticancer agent, Resveratrol (a structural analog of Moscatilin), whose presence has also been reported in *Dendrobiums*. Considering the threatened nature of this genus, crude extracts of a tropical and epiphytic *Dendrobium* species, viz., *Dendrobium ovatum*, prepared from in vitro seedlings were also tested towards cytotoxicity and radiosensitization efficacy. Moscatilin functioned as an effective radiosensitizer at 5 µg/ml along with 1 Gy X-ray and 200 J/m^2^ UV-C radiations. It was also able to perturb cell cycle both at replicative and post-replicative phases with the aforementioned combination. Moscatilin, in unison with radiation, triggered immunogenic death specifically on cancer cells starting from Pyroptosis, terminating in Necroptosis. Moscatilin, when used singly, could evoke immunogenic cell death. Analyses of Damage-Associated Molecular Patterns released during radiation and Moscatilin treatment would aid in ascertaining the mode of cell death. Moscatilin is a potential radiosensitizer and must be tested for preclinical and clinical trials to combat cancer.

**Supplementary Information:**

The online version contains supplementary material available at 10.1007/s13205-021-02827-3.

## Introduction

**Fig. 1:**
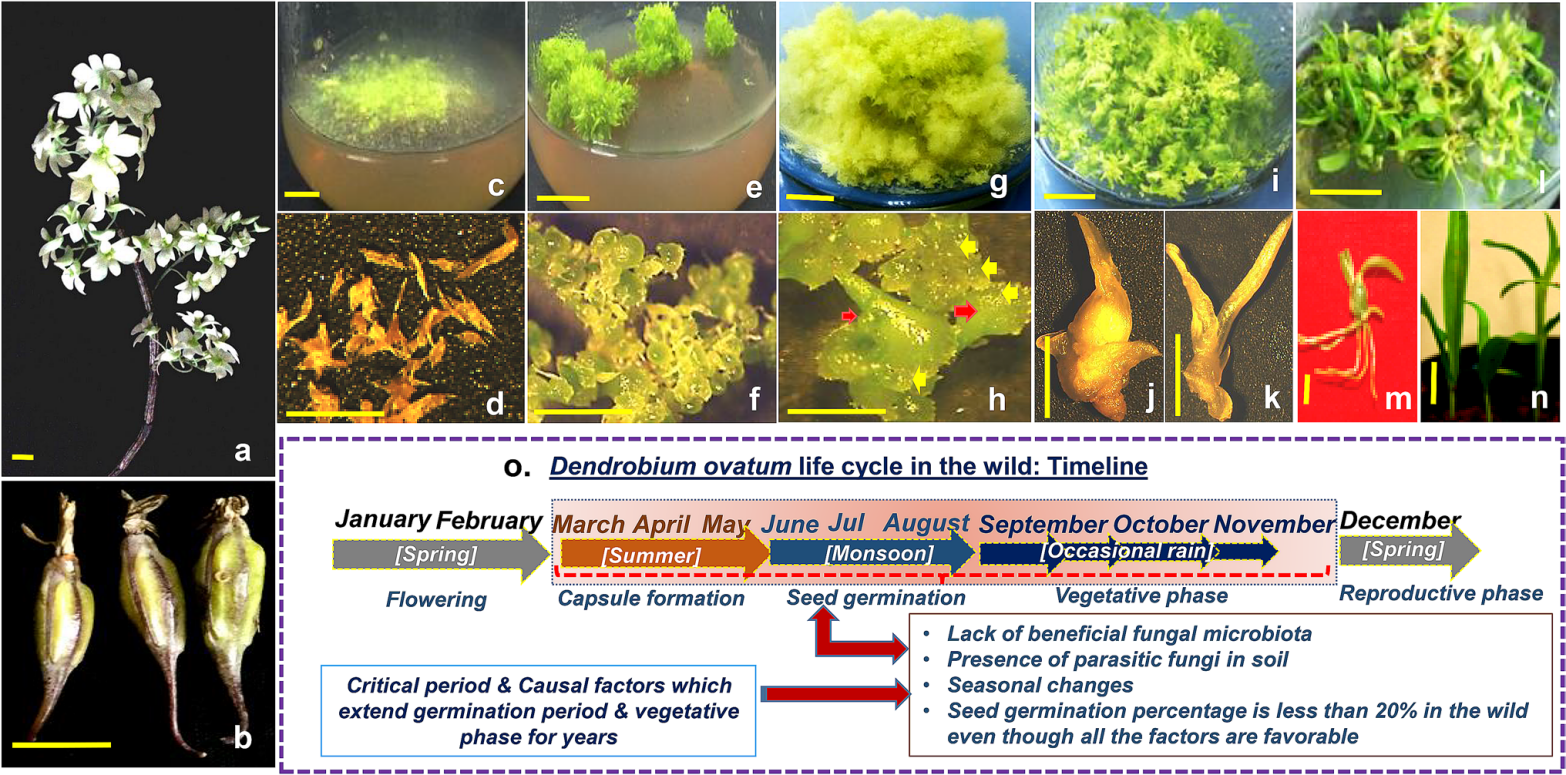
**a** Habit of wild *Dendrobium ovatum* with inflorescence. **b** Capsules (fruits) of *D. ovatum* bearing seeds. **c** Seeds on the half-strength MS basal medium, shown on the 1st day of inoculation (seed culture). **d** Stereomicrographs of seeds. **e** Transition of seeds into spherules on the 10th day. **f** Stereomicrographs of spherules on the 10th day. **g** Hypergeneration of protocorms and protocorm-like bodies (PLBs) in culture on the 40th day. **h** Stereomicrographs of the hypergenerating protocorms (red arrows) with PLBs (yellow arrows), generated through culture on the 40th day. **i** Seedlings developed on the 80th day of culture. **j, k** Stereomicrographs of seedlings developed on the 80th day. **l, m** Plantlets on the 100th day of culture that was chosen for the preparation of crude extract. **n** Acclimatization of in vitro raised plantlets of *D. ovatum* for ecorestoration purposes. **o** Life cycle of *Dendrobium ovatum* in the wild, depicting the critical phases (highlighted in pink) (Scale bar = 1 cm)

Moscatilin is chemically 4,4′-dihydroxy-3,3′,5-trimethoxybibenzyl (also known as, ‘Dendrophenol’, molecular formula: C_17_H_20_O_5,_ molecular weight: 304.34 g/mol) and is mostly present in the genus *Dendrobium*. Moscatilin, isolated from wild *Dendrobiums*, has been shown to display anticancer/antitumor activity against different cancer types (Guan et al. 2019; Cardile et al. 2020), negating the process of metastasis (Pai et al. 2013). Majumder and Sen 1987 isolated this compound for the first time from *Dendrobium moscatum*. A study by Thomas et al. 2017 reported the presence of Resveratrol along with Moscatilin in protoplast cultures of *D. ovatum*. Resveratrol is another significant stilbenoid anticancer agent found in *Dendrobium* species, which is structurally similar to Moscatilin, and both belong to the polyphenol category and possess a bibenzyl structure. Hence, it would be interesting to analyze and compare their efficacy. Additionally, the *Dendrobium* plant extract prepared out of wild samples was also shown to have anticancer, antidiabetic, antihyperglycemic, anti-inflammatory, antioxidant activity, etc. (Teixeira da Silva and Ng 2017). *Dendrobium* is the second largest epiphytic genus of Orchidaceae (Wang et al. 2020), which is popular among the floriculturists. Wild *Dendrobium* species serve as genetic stocks for creating novel ornamental hybrids. However, many more species of *Dendrobium* are yet to be discovered, and some remain underexploited. Their survival is severely threatened (Sarsaiya et al. 2020), especially in the wild tropics, as it is largely dependent on many factors such as (1) availability of abiotic factors [light, temperature, and humidity], which is the underlying cause of their seasonal responses, (2) obtainability of suitable ectomycorrhizal ascomycete or basidiomycete partners for their filamentous seed survival and sprouting, (3) uncongenial edaphic factors in the tropics, which are (a) opulent with parasitic soil-borne fungus, (b) insufficient with organic matter, (c) deficient of potassium content, and (d) scarce in moisture content. Severe exploitation for floriculture purposes and relentless deforestation connected to urbanization are two of the main reasons behind the threatened nature of genus *Dendrobium*. The *Dendrobium* seeds generally tend to sprout in the suitable microsites of aged tree trunks and branches. Species of interest, *Dendrobium ovatum,* commonly known as the ‘green-lipped *Dendrobium*’ (Fig. 1a) is one such tropical epiphytic species, endemic to the Western Ghats, India, which is threatened because of the above-mentioned reasons. This species encompasses a deceptive pollination mechanism like any other orchids (Kamaladhasan et al. 2020). Even though the seeds are well adapted to be dormant during summer, their inherent latency breaking ability is not well studied till date. Very little has been studied about the medicinal properties of *D. ovatum* too in context of the therapeutic secondary metabolites it holds. Considering the threatened position of *D. ovatum*, in this study, we devised a tissue culture propagation method to obtain seedlings of *D. ovatum* through seed culture ensuring its conservation. The extracts prepared from these in vitro seedlings of *D. ovatum* (DOCRE) were also subjected towards studying cytotoxicity, cell death modalities (apoptosis/necrosis), cell-cycle phases, and radiosensitivity in comparison with the same induced by Moscatilin and Resveratrol in cancer cells.

Ionizing radiations are often used for treating many human cancers. However, this radiotherapy mostly demands high radiation doses towards the destruction of cancer cells which in turn cause toxicity in the surrounding healthy cells. The combination treatment of radiation and chemotherapeutic agent was reported to cause more harmful consequences in this regard leading to multiple organ dysfunction (Hazra et al. 2011). Therefore, it is very essential to establish the treatment regimen that uses minimum radiation dose sensitizing only the tumor cells without causing damage to the normal cells. Many phytochemicals have been reported under two different categories, viz., ‘radiosensitizers’ (compounds that selectively make the tumor cells more sensitive towards radiation, with application of an optimum and non-toxic dose for healthy cells) and ‘radioprotectors’ (compounds that offer protection towards the normal cells from damaging radiations) towards safe and efficient cancer radiotherapy (Kuruba and Gollapalli 2018). Many phenolics have been observed with radiosensitizing effects, and they also regulate cell cycle and the process of apoptosis (Tiwari and Mishra 2019). The stilbene, Resveratrol, has been demonstrated to have both radiosensitizing and radioprotecting effects during the therapy, but there is still inadequacy in clinical studies approving the usage of this phytocompound as a chemo-radioprotector/chemo-radiosensitizer (Mortezaee et al. 2020). Despite being structural analogs, the active principle, Moscatilin has only been studied marginally towards its chemo-radiosensitizing/chemo-radioprotecting effects till date. Chen et al. 2019 have reported the radiosensitizing effect of Moscatilin for the first time on two different esophageal cancer cell lines, viz., CE81T/VGH and BE3. There is no study yet signifying the comparative action of both potent anticancer agents, Moscatilin and Resveratrol in chemo-radiotherapy. Apart from it, as both these phytochemicals reside in the threatened genus *Dendrobium*, the study related to the effectiveness of *Dendrobium* crude extract (prepared from in vitro seedlings) towards radiosensitization/protection will also be useful. Comprehensively, this idea forms one of the main basis of the present study, which is intended to delineate and correlate the impact of commercial Moscatilin, Resveratrol, and *Dendrobium ovatum* crude extract (DOCRE), prepared from in vitro seedlings on selected cell lines, exposed to X-ray and UV-C radiations. X-ray has been highly and securely used in the treatment of various cancers (Mohan et al. 2019), whereas the studies on the effect of UV-C radiation towards tumor destruction are limited. As per a research report by Müller et al. 2018, UV-C (range 200–280 nm) has been shown as an efficient radiosensitizer causing tumor cell inactivation in a localized mode along with cell-cycle arrest through oxygen independent photochemistry, without affecting the normal cells. Another study has reported the cancer cell killing capability of a very low dose of UV-C radiation over UVA/UVB (Kimura et al. 2010).

## Materials and methods

### In vitro seed germination and development of seedlings

The *D. ovatum* capsules were first surface sterilized with 10% Tween-20 detergent for 10 min and then were washed under running tap water for 30 min. This was followed by washing the capsules with autoclaved double distilled water two or three times inside the laminar flow cabinet. Then the capsules were treated with 0.1% mercuric chloride for 10 min. After that, the capsules were rinsed with autoclaved double distilled water for five times, blotted dry, cut longitudinally, and the seeds were dusted onto half-strength Murashige & Skoog medium (Murashige and Skoog 1962) with MS macroelements-4.23 g/l, supplemented with 1 mg/l zeatin, 2% sucrose, and the pH was adjusted to 5.8. The medium was solidified with 0.8% agar. The culture bottles were incubated in a sterile condition at a temperature of 25 ± 2 °C with 12-h photoperiod maintained by fluorescent lights (Philips, India) with an intensity of 50 µmol/m^2^/s. The entire life cycle of *D. ovatum *in vitro, from seeds to the developed seedlings, was staged and identified under a stereo zoom microscope (‘Motic’) equipped with the software ‘Motic images plus 2.0’. All the developmental stages were stereomicrographed.

### The ultrasonication low-temperature-based extraction procedure

One gram of young seedling of *D. ovatum* was used for the preparation of crude extract. First, the tissue was macerated to a fine pulp with liquid nitrogen using a mortar and pestle (Alara et al. 2021). Then, around 1–2 ml of the extraction solvent methanol was added to the pulp. The mixture was pulse-sonicated at 40 W (intermittent pulse) for 30–45 min. Throughout the procedure, the samples were kept on ice. The mixture obtained was then centrifuged at 4500 RCF for 30 min at 4 °C. The clear supernatant was concentrated using lyophilization in a very low temperature. The lyophilized powder from one tube is 8 mg, and when the replicates were pooled, we had a total of 80 mg which was sufficient for our assays. The concentration of Moscatilin and Resveratrol in the pooled lyophilized sample of DOCRE came to 5 and 12 µg/g d.wt, respectively. However, the purified Moscatilin, which was ~ 2.5 mg from 60 g of in vitro tissue, was quite insufficient for various experiments, and hence, we used purchased Moscatilin for all assays (Supplementary file). However, the extraction was done to quantify the Moscatilin levels both in in situ and in vitro samples.

### Testing the cytotoxicity of Moscatilin on selected human cancer cell lines

MTT [3-(4,5-dimethylthiazol-2-yl)-2,5,diphenyltetrazolium bromide] assay was performed to find the half maximal inhibitory concentration (IC_50_) of Moscatilin on few significant cancer cell lines such as HepG2, SiHa, HT-29, Saos-2, and Cal-27. Fibroblasts were used as control cells (Supplementary file). The cells were cultured in Dulbecco’s Modified Eagle Medium (DMEM) supplemented with 4 mg/l folate along with penicillin (100 U/ml)-streptomycin (100 µg/ml) combination and were incubated at 5% CO_2_ and 95% humidity at 37 °C. 10% of heat-inactivated FBS was supplemented to the culture medium. The cells were subcultured two times a week, and the doubling time was calculated. Cells were briefly washed with 5 ml of sterile phosphate-buffered saline (PBS) first, incubated for 3 min at 37 °C with 0.25% trypsin–EDTA, and then, the cells were dislodged from the culture flask. Media containing FBS were added to stop the reaction and passaged to a new culture flask for in vitro experiments.

1 × 10^4^ cells were seeded into 96-well plates with DMEM medium-containing 10% FBS for 48 h. Human fibroblast was used as the non-cancerous cell line for comparison in the exhibited effect. Mitomycin-C was used as a positive control to assess the inhibitory potential of Moscatilin. The compound was dissolved in 0.1% dimethyl sulfoxide (DMSO). Three control groups were used for the study. They were: Group 1—control set containing DMEM medium alone (negative control-1); Group 2—control set containing methanol along with DMEM medium (vehicle control-1); Group 3—control set containing 0.1% DMSO along with DMEM medium (vehicle control-2). Once the cells were seeded, they were allowed to adhere to the culture flask for 24 h at 37 °C. The test group cells were incubated with different concentrations (range 1 to 12 µg/ml) of Moscatilin and Mitomycin-C for 48 h, and were washed with PBS.

Cells were post-incubated for 4 h at 37 °C with 100 µl of PBS-dissolved MTT (1 mg/ml). After the PBS wash, cells were lysed using solvents with gentle shaking for 10 min to dissolve the formazan crystals completely. Absorbance values were taken at 570 nm using the Infinite M20 microplate spectrophotometer system (Tecan Instruments, Austria). Each treatment was done in triplicates, and the absorbance given by the untreated cells was taken as hundred percent survivals. The results were normalized with control samples. The IC_50_ values were determined from the concentration versus viability plot.

### Evaluating the cytotoxicity and radiosensitization effects of Moscatilin, Resveratrol, and *Dendrobium ovatum* crude extract (DOCRE) on selected cell lines

For this study, three different cell lines, viz., HepG2, SH-SY5Y, and HaCaT, were chosen. HepG2 and HaCaT were cultivated as monolayer in DMEM supplemented with 10% FBS at 37 °C under a humidified environment with 5% CO_2_, whereas SH-SY5Y was cultivated as monolayer in DMEM/Ham’s F-12 nutrient mixture (ratio 1:1), supplemented with 10% FBS with above-mentioned temperature and CO_2_ conditions. The clonogenic cell survival assay was performed to check the survival rate of X-ray and UV-C irradiated HepG2, SH-SY5Y, and HaCaT cell lines. The cytotoxicity effects of Moscatilin, Resveratrol, and DOCRE were also analyzed on these three cell lines through their individual treatment and their radiosensitization effects were also assessed through combination treatment with X-ray and UV-C radiations as done in a study elsewhere (Chen et al. 2019). The clonogenic assay was used to determine the colony formation after each individual and combination treatment, by staining it with crystal violet dye. When the cells attained 80% confluence, the total cell count and viability assessments were performed using trypan blue staining. Based on the cell count and doubling time, 300 cells/ml were seeded onto 6-well plates (test and control) with triplicates. Growing cells were subjected to the following treatments:

**Set 1**: HepG2, SH-SY5Y, and HaCaT were independently treated with DOCRE in triplicates. The concentrations of DOCRE used were 1, 10, and 12.5 µg/ml.
**Set 2**: HepG2, SH-SY5Y, and HaCaT were separately subjected to X-ray irradiation in triplicates. The radiation was imparted to the cells by the instrument, Faxitron X-ray, 43855F-CP160 Option, Lincolnshire, Illinois, USA. The dosages of X-ray used were 1, 3, and 5 Gy.
**Set 3**: HepG2, SH-SY5Y, and HaCaT were individually subjected to UV-C radiation in triplicates. The radiation was imparted to the cells by the instrument, Stratagene UV Stratalinker 2400. The dosages of UV-C radiation used were 20, 200, and 2000 J/m^2^.
**Set 4**: HepG2, SH-SY5Y, and HaCaT were subjected to the combination treatment of 5 µg/ml DOCRE and the selected dose of X-ray (1 Gy).
**Set 5**: HepG2, SH-SY5Y, and HaCaT were subjected to the combination treatment of 5 µg/ml DOCRE and the selected dose of UV-C (200 J/m^2^).
**Set 6**: HepG2, SH-SY5Y, and HaCaT were independently treated with Moscatilin in triplicates. The concentrations of Moscatilin used were 1, 10, and 12.5 µg/ml.
**Set 7**: HepG2, SH-SY5Y, and HaCaT were subjected to the combination treatment of 5 µg/ml Moscatilin and the selected dose of X-ray (1 Gy).
**Set 8**: HepG2, SH-SY5Y, and HaCaT were subjected to the combination treatment of 5 µg/ml Moscatilin and the selected dose of UV-C (200 J/m^2^).
**Set 9**: HepG2, SH-SY5Y, and HaCaT were independently treated with Resveratrol in triplicates. The concentrations of Resveratrol used were 1, 10, and 12.5 µg/ml.
**Set 10**: HepG2, SH-SY5Y, and HaCaT were subjected to the combination treatment of 5 µg/ml Resveratrol and the selected dose of X-ray (1 Gy).
**Set 11**: HepG2, SH-SY5Y, and HaCaT were subjected to the combination treatment of 5 µg/ml Resveratrol and the selected dose of UV-C (200 J/m^2^).
**Set 12**: HepG2, SH-SY5Y, and HaCaT were independently treated with Mitomycin-C in triplicates. The concentrations of Mitomycin-C used were 1, 10, and 12.5 µg/ml.
**Set 13**: HepG2, SH-SY5Y, and HaCaT were subjected to the combination treatment of 5 µg/ml Mitomycin-C and the selected dose of X-ray (1 Gy).
**Set 14**: HepG2, SH-SY5Y, and HaCaT were subjected to the combination treatment of 5 µg/ml Mitomycin-C and the selected dose of UV-C (200 J/m^2^).

All treatment series for every cell line had exclusive untreated control plates in triplicates. Cell survival capacity and cell death were observed by imaging through an inverted phase-contrast microscope (Inverted microscope Zeiss AxioVert.A1, phase contrast). The survival rate of the cells was estimated through colony count in the form of plating efficiency (PE) in terms of percentage (%) and surviving fraction (SF). PE was calculated as, no. of colonies formed/no. of cells seeded × 100 and SF was estimated as, no. of colonies formed after treatment/(no. of cells seeded × PE).

### Scrutinizing the cell-cycle perturbations triggered by Moscatilin, Resveratrol, and DOCRE treatments

Cell-cycle analysis was carried out to determine the percentage of cells in different sub-phases after 72 h. The treatment groups included (1) UV-C radiation [200 J/m^2^] treated cells, (2) X-ray [1 Gy] irradiated cells, (3) cells treated with DOCRE [5 µg/ml], (4) cells treated with Moscatilin [5 µg/ml], (5) cells treated with Resveratrol [5 µg/ml], and (6) Mitomycin-C [5 µg/ml] treated cells. The combination of a drug [5 µg/ml] with UV-C radiation [200 J/m^2^] and X-ray radiation [1 Gy] were also assessed. For this analysis, first, all the three cell lines were cultivated at 37 °C under a humidified environment with 5% CO_2_. When the cells attained 80% confluency, they were trypsinized and seeded in 6 cm^2^ Petri plates at a cell density of 2 × 10^5^ cells/plate. The plates were incubated for 48 h then. After this, DMEM medium was aspirated, and the Petri plates were washed with PBS (2 ml) twice. Approximately 200 µl of PBS was retained in the plates for radiation treatments. The cells were then exposed to treatment with radiations. In the plates for treatment with Moscatilin, Resveratrol, DOCRE, and Mitomycin-C, 3 ml of media was used, after which the drugs were added alone or, in combination. The control plates were left untreated and all the plates were incubated for another 72 h. Post 72 h, the media was decanted, and the cells were washed with PBS (2 ml) twice. The plates were trypsinized, and PBS (1 ml) was added. The cells were then collected in a falcon tube and centrifuged at 1800 RCF for 10 min. The pellet was resuspended in 70% alcohol and incubated at room temperature for 10 min. Then, the samples were again subjected to centrifugation at 1800 RCF for 10 min. The pellet was incubated with ribonuclease (RNase) in a water bath at 37 °C for 1 h. After incubation, 10 µl of propidium iodide (PI) was added and kept for 10 min at room temperature. The samples were then diluted with PBS and analyzed through flow cytometry using BD FACSCalibur (BD Biosciences, San Jose, CA, USA), and the data obtained were analyzed using BD CellQuest Pro software (BD Biosciences). Cell population was gated in the scatter plot, and using a linear scale, cell cycle was observed. Cell count vs. PI plot was analyzed to visualize various cells in different phases of the cell cycle. DNA content in the untreated and treated systems of every cell line was plotted as frequency histograms, that indicated the relative percentage of cells in various cell-cycle phases (G0/G1, S, and G2/M).

### Assessment of the modes of cell death (apoptosis/necrosis) caused by Moscatilin, Resveratrol, and DOCRE

For accessing the cell death modes, in this research study, Annexin V-Fluorescein isothiocyanate (FITC)/PI staining was performed using FITC Annexin V Apoptosis detection kit I, BD Pharmingen™. The kit contained three different components, viz., 10X Annexin V binding buffer, FITC Annexin V, and PI staining solution. This analysis was carried out chiefly to detect the apoptotic cell death (early and late) and also necrosis within a heterogeneous cell population, in the studied cell lines. Before the analysis, all the cell lines were cultured and treated as per the previously mentioned protocol. The treatment groups were also same as the ones used for cell-cycle analysis. After 72 h of treatment, the medium was discarded from the plates and the treated cells were washed with 2 ml of PBS two times. Post-wash, the cells were trypsinized and 1 ml of cold PBS was added. Cells were then collected in a falcon conical centrifuge tube and centrifugation was done at 1000 RCF for 10 min. After centrifugation, the pellet was resuspended in 100 µl volume of 1X binding buffer. This was followed by the addition of 5 µl volume of both FITC Annexin V and PI to the falcon tube except the control. The tubes were then vortexed for 5 min. After vortexing, the cells were incubated in dark at 25 ℃ for 15 min. After incubation, 1X binding buffer was added to the tube and the volume was made up to 500 µl. This was followed by processing the samples through flow cytometry (BD FACSCalibur, BD Biosciences, San Jose, CA, USA) and the data obtained were analyzed using BD CellQuest Pro software (BD Biosciences). The generated two-dimensional dot plots indicated percentage of unaffected cells [lower left quadrant − (Annexin V^−^/PI^−^)], early apoptotic cells [lower right quadrant − (Annexin V^+^/PI^−^)], late apoptotic cells [upper right quadrant − (Annexin V^+^/PI^+^)], and necrotic cells [upper left quadrant − (Annexin V^−^/PI^+^)].

### Statistical analysis

All the data were presented in the form of mean ± standard deviation from three independent experiments. Statistical significance differences were computed using Student’s *t* tests by GraphPad Prism 8.0, where values with *P < 0.05, **P < 0.01, ***P < 0.001, and ****P < 0.0001 were considered statistically significant and ‘ns’ depicted non-significant at the 0.05 probability level.

## Results and discussion

### Significance of seed propagation in *Dendrobium ovatum*

For seed culture, the *D. ovatum* capsules (Fig. 1b) were used. Seeds were taken out from these capsules and inoculated onto half-strength MS culture medium, supplemented with 1 mg/l Zeatin (Fig. 1c, d). These seeds transformed into spherules (Fig. 1e, f), which displayed signs of both somatic and zygotic embryogenesis (Fig. 1g). These spherules gradually developed into both protocorms and protocorm-like bodies (PLBs) (Fig. 1h). The generation of young seedlings (Fig. 1i–l) both from protocorms and PLBs was witnessed during the culture, and eventually, they grew into plantlets with well-developed roots and shoots in just 3 months (Fig. 1m, n). In the wild habitat, 100% *Dendrobium* seed survival is almost impossible, because of the protracted mycorrhizal presence favoring germination. In the nature, *D. ovatum* seeds are always dependent on species-specific fungal hyphae and this delays the life cycle of this tropical orchid species for months, sometimes even years. This in vitro culture platform has hastened the life cycle of *D. ovatum* species (Fig. 1o) to tide over the difficulties faced during the vegetative phase. Protocorms formed during the *D. ovatum* life cycle in vitro were tubular structures and they are related to zygotic embryogenesis (Cardoso et al. 2020). However, during tissue culture, apart from this protocorm development, the secondary and tertiary protocorm-like structures termed as protocorm-like bodies (PLBs) also formed from the pre-existing protocorms. These PLBs differ from protocorms in their ontogeny. They are either developed from the meristemoids of explants or, the callus. In the present study, the explants used were filamentous seeds, which developed into protocorms and each protocorm could develop PLBs from their meristemoids. Since the initiation of PLBs is from meristemoids of somatic tissues, they are a sequel of somatic embryogenesis, which are only witnessed across in vitro cultures. Therefore, in vitro propagation was not only congenial for *D. ovatum* seed survival, but also for amplified hypergeneration of seedlings/plantlets through protocorm and PLB formation. Half-strength MS medium along with zeatin supplementation was proven to be very beneficial towards the seed germination in *D. ovatum* like other *Dendrobiums* (Teixeira da Silva et al. 2015). In situ conditions lack several intrinsic benefits of plant tissue culture, such as the culture platform ensures precise control on growth conditions and batch-to-batch product consistency without any fungal hyphae involvement (i.e., asymbiotic mode). The principal compound of interest, Moscatilin, is gaining medicinal value lately. Chemical synthesis of this compound remains unattempted and currently, researchers and herbal drug makers are still relying on in situ bioresources for isolation of essential stilbenoids, including Moscatilin. The species of interest, *D. ovatum*, and its pollination mechanisms are not well studied yet. This plant is listed as threatened, considering the rarity in population distributions. The conservation status of this species poses a hindrance to collection policies. Therefore, the conservation of *D. ovatum* through tissue culture strategy becomes inevitable towards the study of medicinal compounds like Moscatilin and Resveratrol. The seedlings obtained through this culture approach were subjected towards liquid nitrogen grinding, followed by ultrasonication and lyophilization. All these procedures were proven successful in giving a concentrated extract of *D. ovatum*, that was utilized for cytotoxicity and radiosensitization studies.

### Cytotoxicity induced by Moscatilin as confirmed through MTT assay

**Fig. 2:**
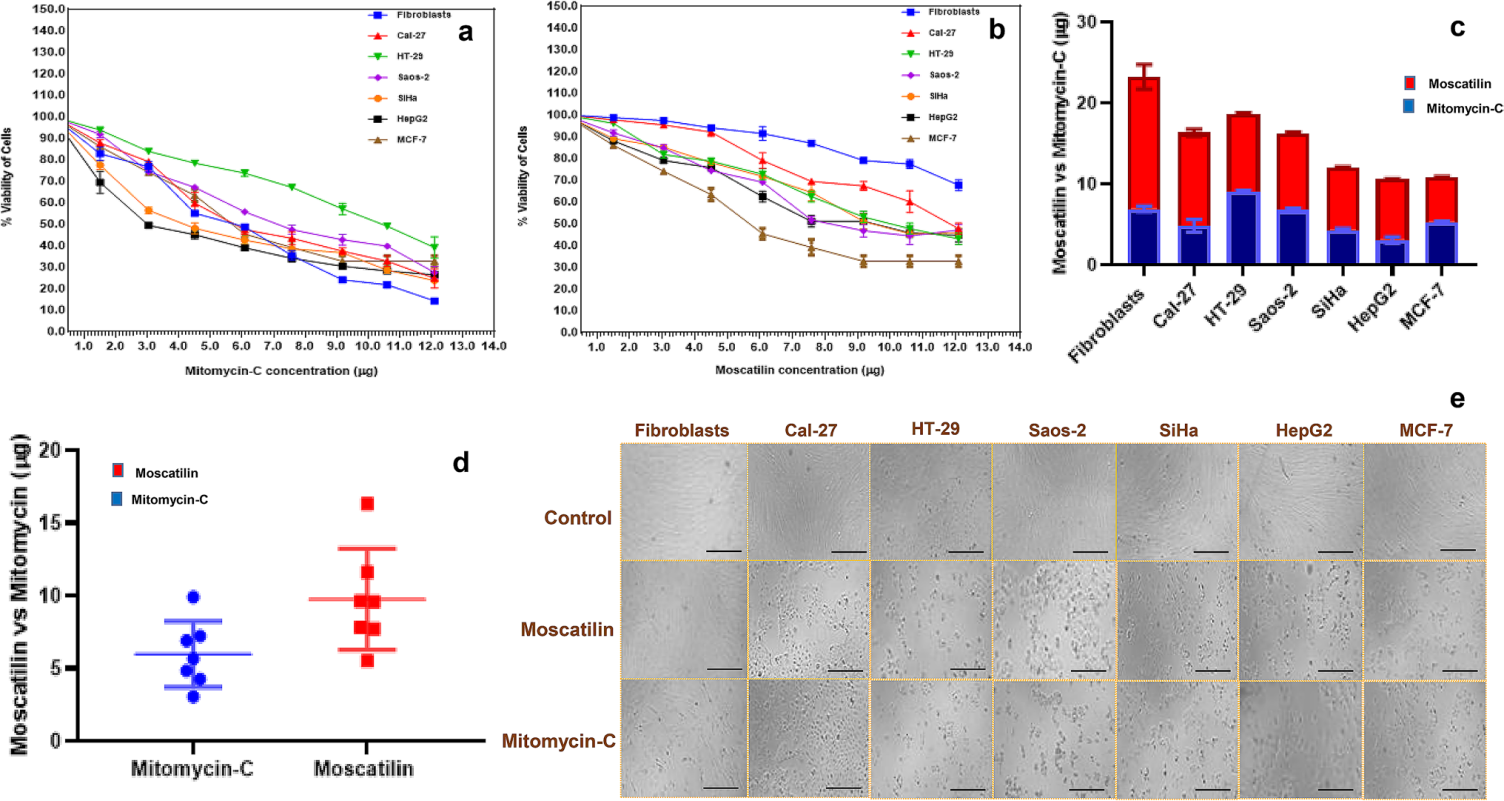
Implication of cell viability percentage after 48 h, showing the efficacy of** a** Mitomycin-C. **b** Moscatilin. **c, d** Comparison of IC_50_ (µg) of Mitomycin-C and Moscatilin (blue bars represent Mitomycin-C, and red bars represent Moscatilin). **e** Phase-contrast microscopic images of the studied cell lines in 96-well microplates (MTT assay), displaying control group and test (Moscatilin treated and Mitomycin-C treated) groups. (Scale bar = 100 µm)

The IC_50_ values of Mitomycin-C in the studied cell lines ranged from 2.6 to 10.35 µg/ml (Fig. 2a, d), and the same for Moscatilin ranged from 5.4 to 18 µg/ml (Fig. 2b, d). Mitomycin-C was used as a positive control towards the comparison in cytotoxic effect caused by Moscatilin, as it is a generic drug that is widely used as an antitumor-antibiotic for the treatment of various cancer types. The photomicrographs of the cell lines (Fig. 2e) along with the observations related to viability test and IC_50_ value of Mitomycin-C (Fig. 2c) indicated that it is highly cytotoxic even for fibroblasts (IC_50_ value = 7.2 µg/ml). However, Moscatilin was relatively less cytotoxic to fibroblasts (IC_50_ value = 18 µg/ml) in comparison with Mitomycin-C. The MTT cell proliferation assay indicated that Moscatilin was much more a specific drug towards cancer cell lines and was less harmful to the healthy cells. Both HepG2 and MCF-7 cells were found to be very sensitive towards Moscatilin and Mitomycin-C. SiHa cell lines were highly sensitive to Mitomycin-C (IC_50_ value = 4.5 µg/ml) and were found to be moderately sensitive to Moscatilin (IC_50_ value = 7.85 µg/ml). Among the studied cancer cell lines, Cal-27 was found to be least sensitive to Moscatilin (IC_50_ value = 11.8 µg/ml), but could induce cytotoxicity. HT-29 cell line was least sensitive to Mitomycin-C (IC_50_ value = 10.35 µg/ml).

### Cytotoxic effects and radiosensitization efficacy of Moscatilin, Resveratrol, and DOCRE

**Fig. 3:**
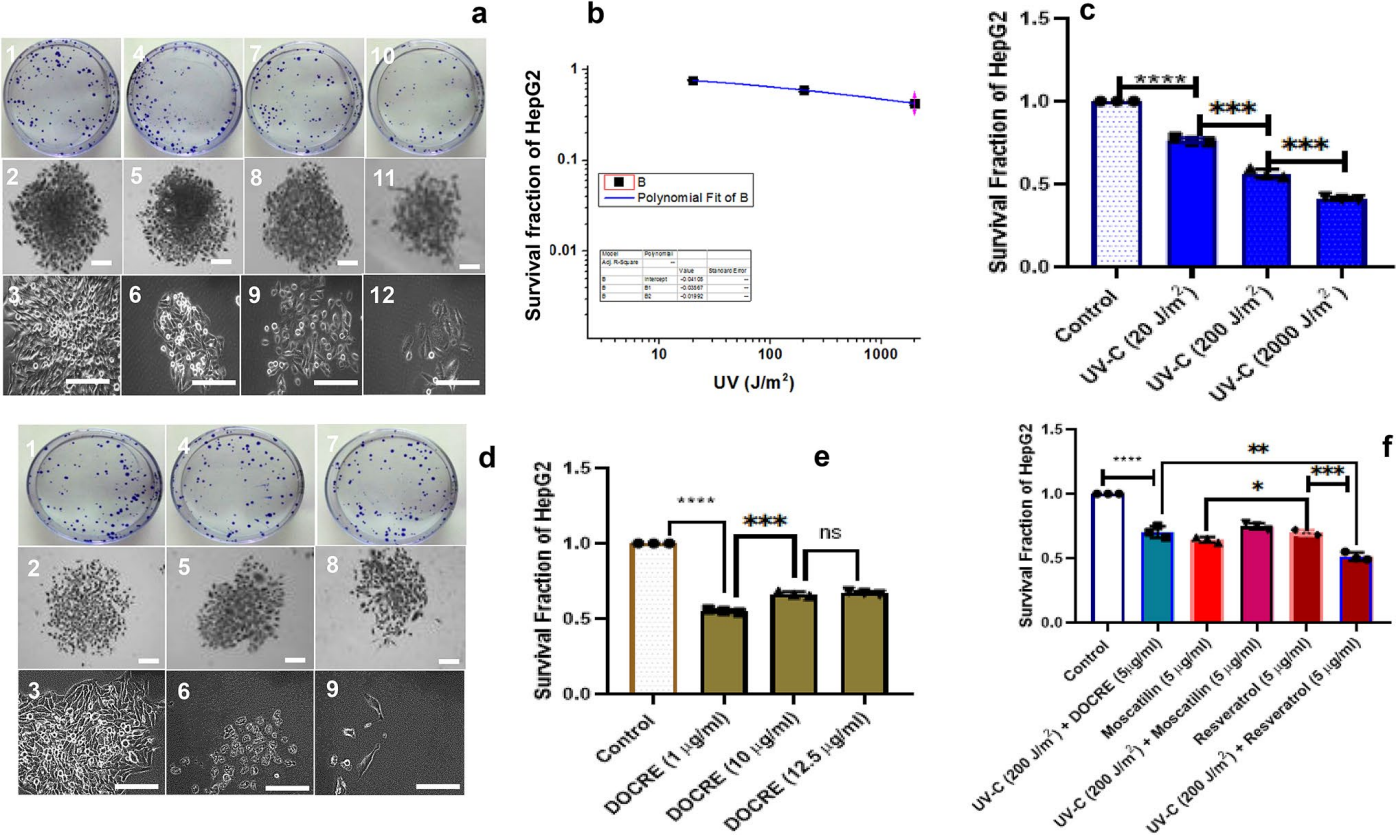
Clonogenic assay performed in HepG2 cell line using various treatments with UV-C radiation, *Dendrobium ovatum* crude extract (DOCRE), Moscatilin and Resveratrol [alone & in combinations]. **a** First, second, third, and fourth columns indicate control, UV-C 20 J/m^2^, UV-C 200 J/m^2^, and UV-C 2000 J/m^2^ treatments, respectively. (1, 2, and 3) HepG2 colonies and cells in control Petri plates, Stereomicrographs of colonies, phase-contrast microscopic image of cells. (4, 5, and 6) HepG2 colonies and cells in UV-C 20 J/m^2^ treated Petri plates, Stereomicrographs of colonies, phase-contrast microscopic image of cells. (7, 8, and 9) HepG2 colonies and cells in UV-C 200 J/m^2^ treated Petri plates, Stereomicrographs of colonies, phase-contrast microscopic image of cells. (10, 11, and 12) HepG2 colonies and cells in UV-C 2000 J/m^2^ treated Petri plates, Stereomicrographs of colonies, phase-contrast microscopic image of cells. **b, c** Survival curve and survival fractions plotted for HepG2 cell line after treatment with three wider doses of UV-C radiations (20 J/m^2^, 200 J/m^2^, and 2000 J/m^2^). **d** First, second and third columns indicate DOCRE 1 µg/ml, DOCRE 10 µg/ml, and DOCRE 12.5 µg/ml treatments, respectively. (1, 2, and 3) HepG2 colonies and cells in DOCRE 1 µg/ml treated Petri plates, Stereomicrographs of colonies, phase-contrast microscopic image of cells. (4, 5, and 6) HepG2 colonies and cells in DOCRE 10 µg/ml treated Petri plates, Stereomicrographs of colonies, phase-contrast microscopic image of cells. (7, 8, and 9) HepG2 colonies and cells in DOCRE 12.5 µg/ml treated Petri plates, Stereomicrographs of colonies, phase-contrast microscopic image of cells. **e** Survival fractions plotted for HepG2 cell line treated with three different concentrations of DOCRE (1 µg/ml, 10 µg/ml, and 12.5 µg/ml). **f** Comparison of survival fractions of HepG2 cell line after treatments with minimum effective doses of UV-C (200 J/m^2^) in combination with drugs, viz., Moscatilin (5 µg/ml), Resveratrol (5 µg/ml) and DOCRE (5 µg/ml). Data presented as Mean ± SD with ****P < 0.0001, ***P < 0.001, **P < 0.01, *P < 0.05 and *ns* non-significant. Scale bar = 100 µm

**Fig. 4:**
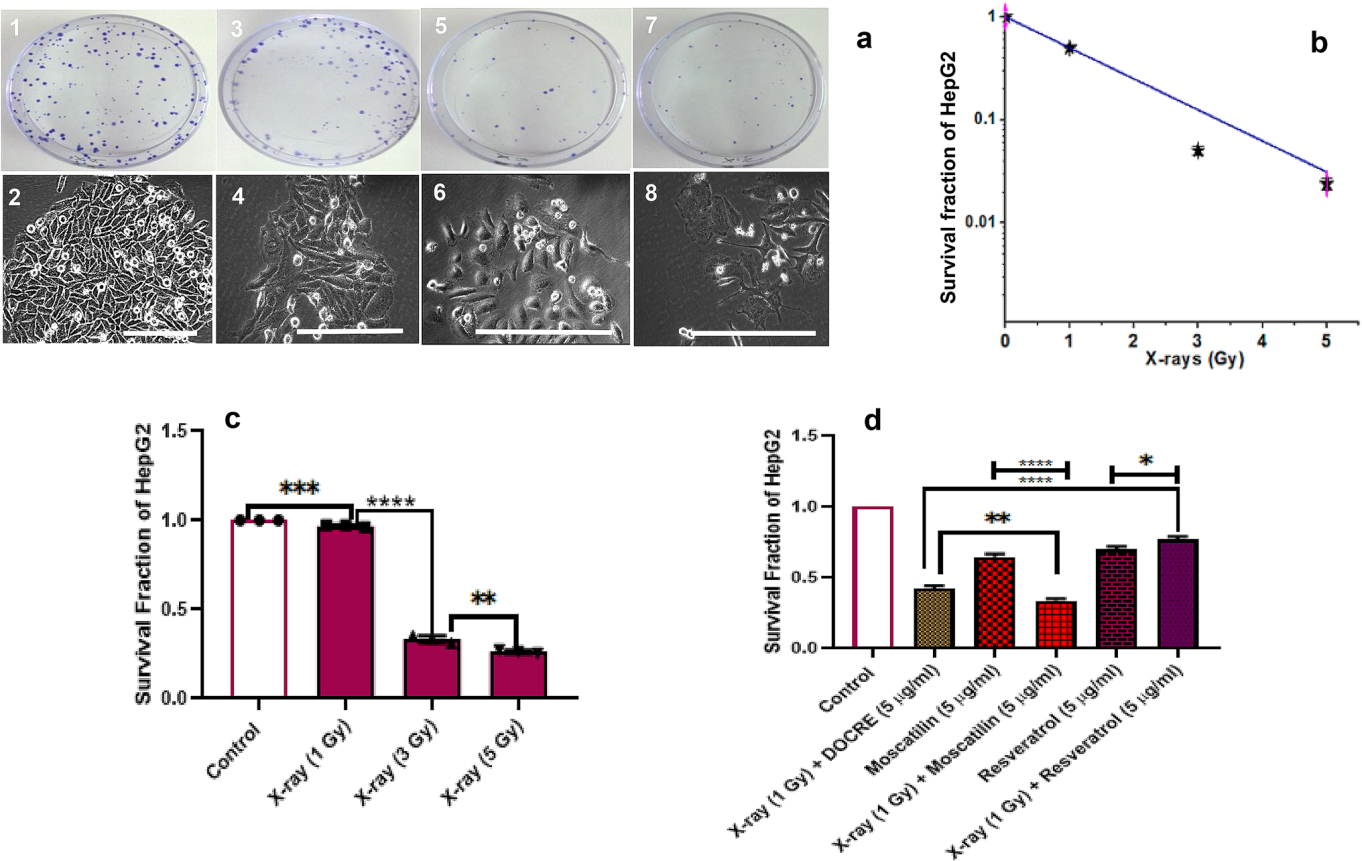
Clonogenic assay performed in HepG2 cell line using various treatments with X-ray radiation, *Dendrobium ovatum* crude extract (DOCRE), Moscatilin, and Resveratrol [alone and in combinations]. **a** First, second, third, and fourth columns indicate control, 1 Gy X-ray, 3 Gy X-ray, and 5 Gy X-ray treatments, respectively. (1, 2) HepG2 colonies and cells in control Petri plates, phase-contrast microscopic image of cells. (3, 4) HepG2 colonies and cells in 1 Gy X-ray treated Petri plates, phase-contrast microscopic image of cells. (5, 6) HepG2 colonies and cells in 3 Gy X-ray treated Petri plates, phase-contrast microscopic image of cells. (7, 8) HepG2 colonies and cells in 5 Gy X-ray treated Petri plates, phase-contrast microscopic image of cells. **b, c** Survival curve and survival fractions plotted for HepG2 cell line after treating with three doses of X-ray radiations (1 Gy, 3 Gy, and 5 Gy). **d** Comparison of survival fractions in HepG2 cell line after treatments with minimum effective doses of X-ray (1 Gy) in combination with drugs, viz., Moscatilin (5 µg/ml), Resveratrol (5 µg/ml), and DOCRE (5 µg/ml). Data presented as Mean ± SD with where ****P < 0.0001, ***P < 0.001, **P < 0.01, and *P < 0.05. Scale bar = 100 µm

The radio-protective/sensitizing effect of Moscatilin has not been much studied till date. The ability of cancer cells to undergo self-duplication and create a colony after treatment (be it a drug, radiation or combined) can be well interpreted using clonogenic assay. This assay takes into account two fundamental measurements to quantitatively assess the effectiveness and the validity of the treatment. One is ‘plating efficiency’, and the other is ‘surviving fraction’. In this research study, a single minimum dose of the drug was chosen after assessing the preliminary tests for clonogenic assay. 5 µg/ml was the chosen concentration for the drugs, viz., Moscatilin, Resveratrol, and Mitomycin-C along with DOCRE. The radiosensitization efficacy testing through clonogenic assay in the present study was restricted to three cell lines, viz., HepG2, SH-SY5Y, and HaCaT. The plating efficiency and surviving fraction calculations of these three cell lines showed that a minimum of 200 J/m^2^ of UV-C (Fig. 3) and 1 Gy of X-ray (Fig. 4) would be the suitable dose of radiation for all the three cell lines. The present study mostly aimed to evaluate, whether both UV-C and X-ray show enhanced inhibition capacity on cancer cells when combined with the main drug of interest, Moscatilin.

**Fig. 5:**
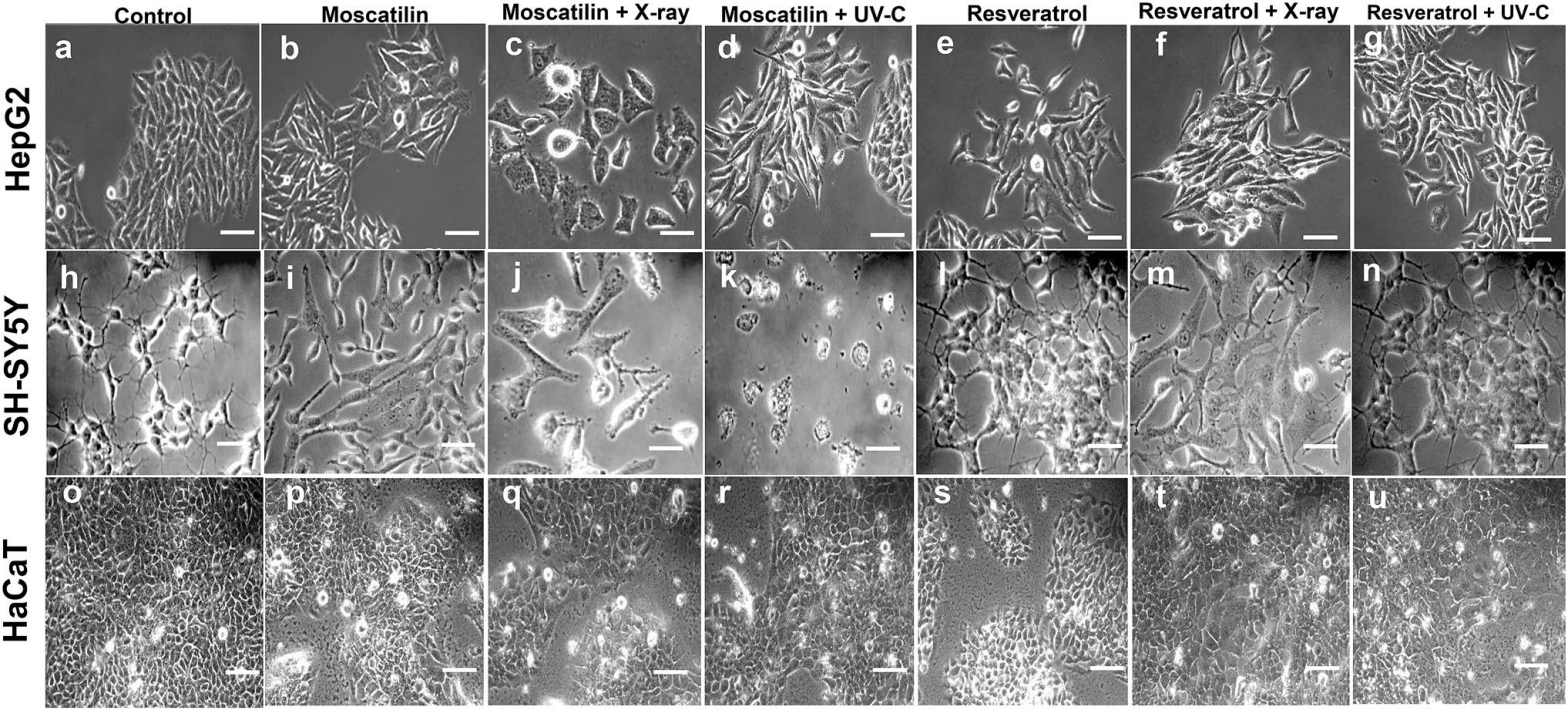
Morphological features of the three studied cell lines (HepG2, SH-SY5Y, and HaCaT) after treatments with Moscatilin and Resveratrol (alone and in combination with minimum effective dose of X-ray and UV-C). **a**–**g** depicts HepG2 cells after 72 h of treatments. **h**–**n** depicts SH-SY5Y cells after 72 h of treatments. **o**–**u** depicts HaCaT cells after 72 h of treatments. The selected X-ray dose was 1 Gy, and that of UV-C was 200 J/m^2^. Rows indicate the cell line type, and the columns indicate different treatments. Scale bar = 100 µm

**Fig. 6:**
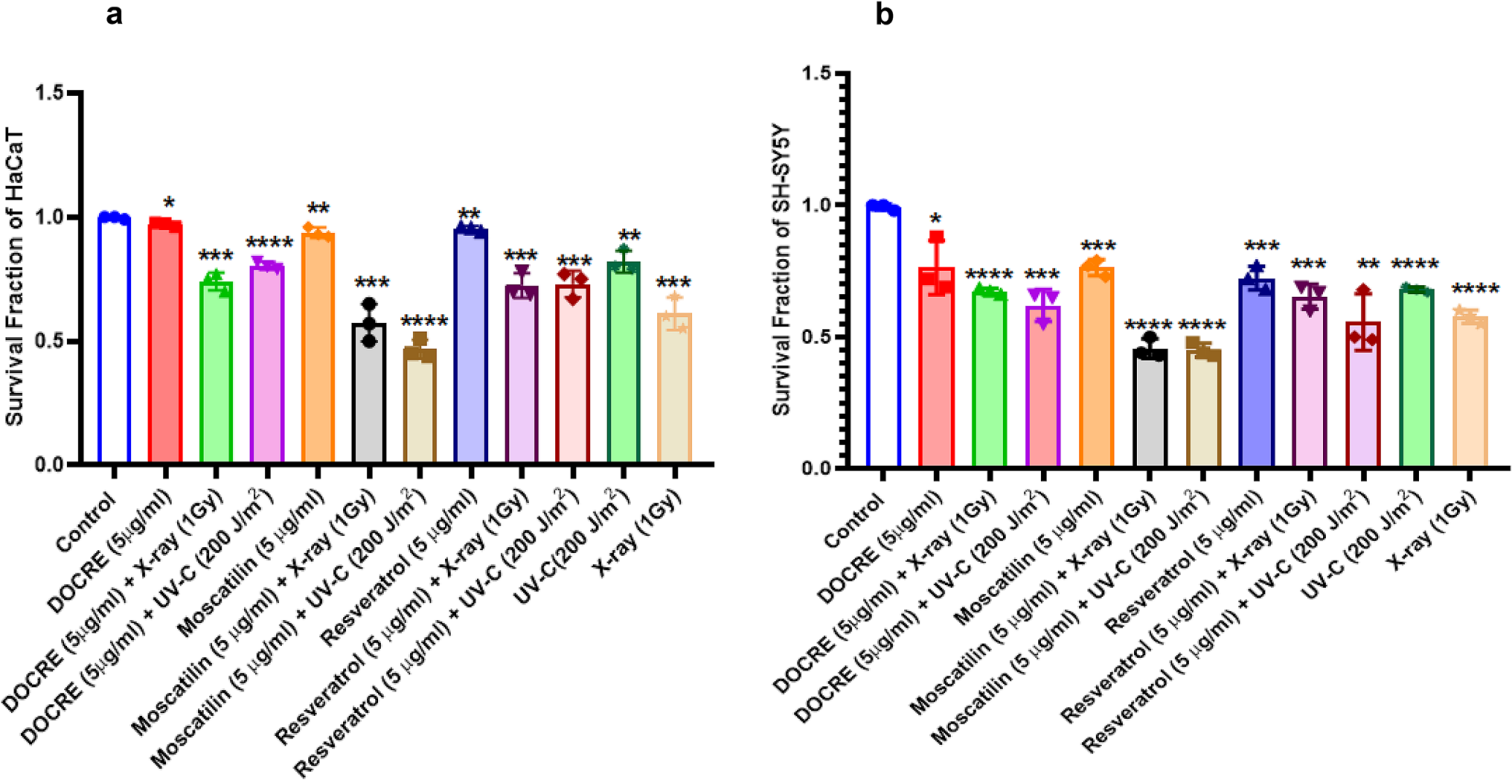
Comparison of survival fractions in HaCaT and SH-SY5Y cell lines under various treatments of X-ray and UV-C radiation along with Moscatilin, Resveratrol, and *Dendrobium ovatum* crude extract (DOCRE) [alone and in combinations]. **a** Graphical representation of survival fractions attained by HaCaT cells after treatment using 1 Gy X-ray and 200 J/m^2^ UV-C radiation in combination with drugs, viz., Moscatilin (5 µg/ml), Resveratrol (5 µg/ml) and DOCRE (5 µg/ml). **b** Graphical representation of survival fractions attained by SH-SY5Y cells after treatment using 1 Gy X-ray and 200 J/m^2^ UV-C radiation in combination with drugs viz., Moscatilin (5 µg/ml), Resveratrol (5 µg/ml), and DOCRE (5 µg/ml). Data presented as Mean ± SD, with ****P < 0.0001, ***P < 0.001, **P < 0.01, and *P < 0.05

The graph related to the surviving fraction of HepG2 cells after UV-C treatment in three wide doses (20 J/m^2^, 200 J/m^2^, and 2000 J/m^2^) indicated a significant decrease in cancer cell survival with the increase in radiation dose (Fig. 3a, c). Whereas, the graph associated with the surviving fraction of HepG2 cells after DOCRE treatment in three different concentrations (1, 10, and 12.5 µg/ml) indicated a significant decrease in cancer cell survival in all the three concentrations as compared to the control group. DOCRE of concentration 1 µg/ml was found to be more effective with a lesser cell survival rate (Fig. 3d, e). When the minimum effective doses (UV-C: 200 J/m^2^ and drugs: 5 µg/ml) were chosen, the combination of (Resveratrol + UV-C) was found to be most effective in decreasing the cancer cell survival. The combined treatment of (Moscatilin + UV-C) and (DOCRE + UV-C) was uniformly effective (Fig. 3f). Individual treatment of Moscatilin and Resveratrol was also found equally effective on HepG2 cells. The surviving fraction graph of HepG2 cells after X-ray irradiation in three doses (1, 3, and 5 Gy) also showed a marked significant decrease in cancer cell survival with the increase in radiation dose (Fig. 4a, c). When the minimum effective doses (X-ray: 1 Gy and drugs: 5 µg/ml) were chosen, the combination of (Moscatilin + X-ray was found to be most effective towards lessening cancer cell survival, followed by the combination treatment of (DOCRE + X-ray) and then (Resveratrol + X-ray) (Fig. 4d). These results have inferred that Moscatilin is a better anticancer agent than Resveratrol on HepG2 cells in combination with X-ray radiotherapy and has good radiosensitization effect. DOCRE and Resveratrol were found to be radio-protective in function. Mitomycin-C treatment was the most deleterious, and no colonies were developed in any of the plates either alone or in combination with radiations (data not shown). It was evident from the cellular features of the colony that, when the cells were treated with X-ray doses, they displayed more prominent cellular shrinkage (Fig. 4a) as compared to the UV-C treatment (Fig. 3a). Plasma membrane blebbing and chromatic condensation was also observed during the X-ray treatment, that has indicated apoptosis (Fig. 4a). The presence of necrotic cells were more clear during the X-ray treatment too, that has implied the transition of apoptosis to necrosis afterwards. SH-SY5Y was observed as the most sensitive cancer cell line towards all the treatments in comparison with HepG2 and HaCaT cell lines (Fig. 5). Combination treatment of (Moscatilin + X-ray) was found strongly effective in killing the cancer cells with features of apoptosis, followed by necrosis in all the three cell lines, as compared to the individual Moscatilin treatments. UV-C in combination with drugs mostly did not have much of a stronger killing effect than X-ray in all the three studied cell lines. Moscatilin was observed as a better radiosensitizer than Resveratrol (Fig. 5). The survival fraction graphs related to HaCaT and SH-SY5Y cell lines also indicated that Moscatilin is a better radiosensitizer as compared to both Resveratrol and DOCRE even at a low concentration of 5 µg/ml. With this radiosensitization potency, it caused more cancer cell destructions in comparison with the individual treatments of Moscatilin, X-ray, and UV-C (Fig. 6).

### Cell-cycle perturbations induced by Moscatilin, Resveratrol, and DOCRE

**Fig. 7:**
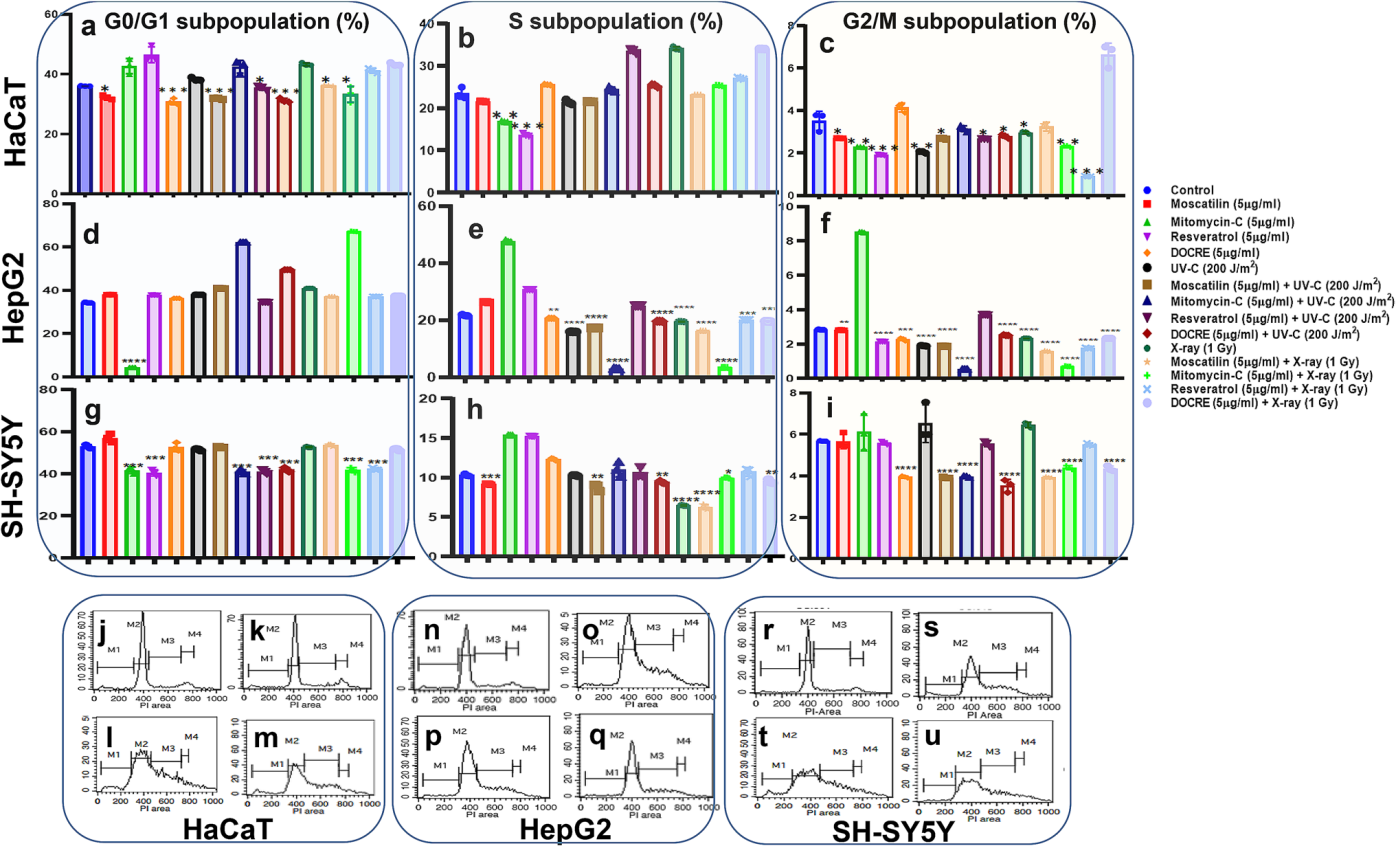
Cell-cycle analyses displaying the cell sub-populations [values represented as percentages] in three studied cell lines, viz., HaCaT, HepG2, and SH-SY5Y under various treatments with X-ray and UV-C radiation, along with Moscatilin, Resveratrol, and *Dendrobium ovatum* crude extract (DOCRE) [alone and in combinations]. **a**–**c** Graphical representation of sub-populations in HaCaT cells at G0/G1, S, and G2/M phases, after various treatments. **d**–**f** Graphical representation of sub-populations in HepG2 cells at G0/G1, S and G2/M phases, after various treatments. **g**–**i** Graphical representation of sub-populations in SH-SY5Y cells at G0/G1, S, and G2/M phases, after various treatments. All the treatments groups are color-coded. CellQuest Pro software generated histograms showing HaCaT cells at different cell-cycle phases in **j** control, **k** treated with 5 µg/ml Moscatilin, **l** treated with 5 µg/ml Moscatilin along with 200 J/m^2^ UV-C, and **m** treated with 5 µg/ml Moscatilin along with 1 Gy X-ray; HepG2 cells at different cell-cycle phases in **n** Control, **o** treated with 5 µg/ml Moscatilin, **p** treated with 5 µg/ml Moscatilin along with 200 J/m^2^ UV-C, and **q** treated with 5 µg/ml Moscatilin along with 1 Gy X-ray; SH-SY5Y cells at different cell-cycle phases in **r** Control, **s** treated with 5 µg/ml Moscatilin, **t** treated with 5 µg/ml Moscatilin along with 200 J/m^2^ UV-C, and **u** treated with 5 µg/ml Moscatilin along with 1 Gy X-ray. M1, M2, M3, and M4 in the histograms **j**–**u** stand for cell population gated for sub-G0, G0/G1, S, and G2/M phases, respectively. Data presented as Mean ± SD with ****P < 0.0001, ***P < 0.001, **P < 0.01, and *P < 0.05 vs control group

To study the inhibitory effects of Moscatilin and other drug/radiation treatments, cell-cycle phases that are linked to cell growth and survival were studied. The tumor cell growth is tightly linked to the cell cycle. The sub-populations of cells in percentage would help us to estimate at which phase the inhibition is remarkable. Moscatilin, when used along with UV-C and X-ray radiations, it was found inhibiting both replicative (S-phase) (Fig. 7b, e, h) and post-replicative (G2/M) phases (Fig. 7c, f, i). Moscatilin causing G2/M phase arrest in the cell cycle aligns with several studies (Ho and Chen 2003; Chen et al. 2008). The present study also aligns with the research reports which stated that Moscatilin induces mitotic catastrophe (Chen et al. 2013) and the effect of the drug increases, when used along with UV-C and X-ray. Both UV-C radiation and ionizing radiation such as X-ray are capable of inducing genotoxic stress-causing mitotic catastrophe (Chen et al. 2019; Fujimoto et al. 2020). Resveratrol, Mitomycin-C, and DOCRE, alone and in combinations with X-ray, and UV-C was able to hinder the G0/G1 phase of the cell cycle in HaCaT cell line (Fig. 7a). The marked inhibition exerted by Moscatilin and DOCRE, alone and in combination with radiation in vitro was able to regulate the proliferation of HaCaT. SH-SY5Y (Fig. 7g–i) appeared to be more sensitive to treatments when compared to HepG2 (Fig. 7d–f) and HaCaT (Fig. 7a–c) as the treatments affected the cells in multiple sub-phases of the cell cycle. Moscatilin in combination with X-ray and UV-C caused cell-cycle arrest even at the G0/G1 phase in HepG2 cells (Fig. 7n–q) and HaCaT (Fig. 7r–u). The G2/M halt by the treatments indicated that the cell-cycle progression is halted before the mitotic entry, preventing the segregation of damaged chromosomes. However, cancer cells have brilliant mechanisms to revert DNA damage induced by radiation. Hence, combinatorial therapy with effective radiosensitizers like Moscatilin at a low and non-toxic concentration might help in specific destruction of cancer cells without affecting the normal cells.

### Mode of cell death induced by Moscatilin, Resveratrol, and DOCRE

**Fig. 8:**
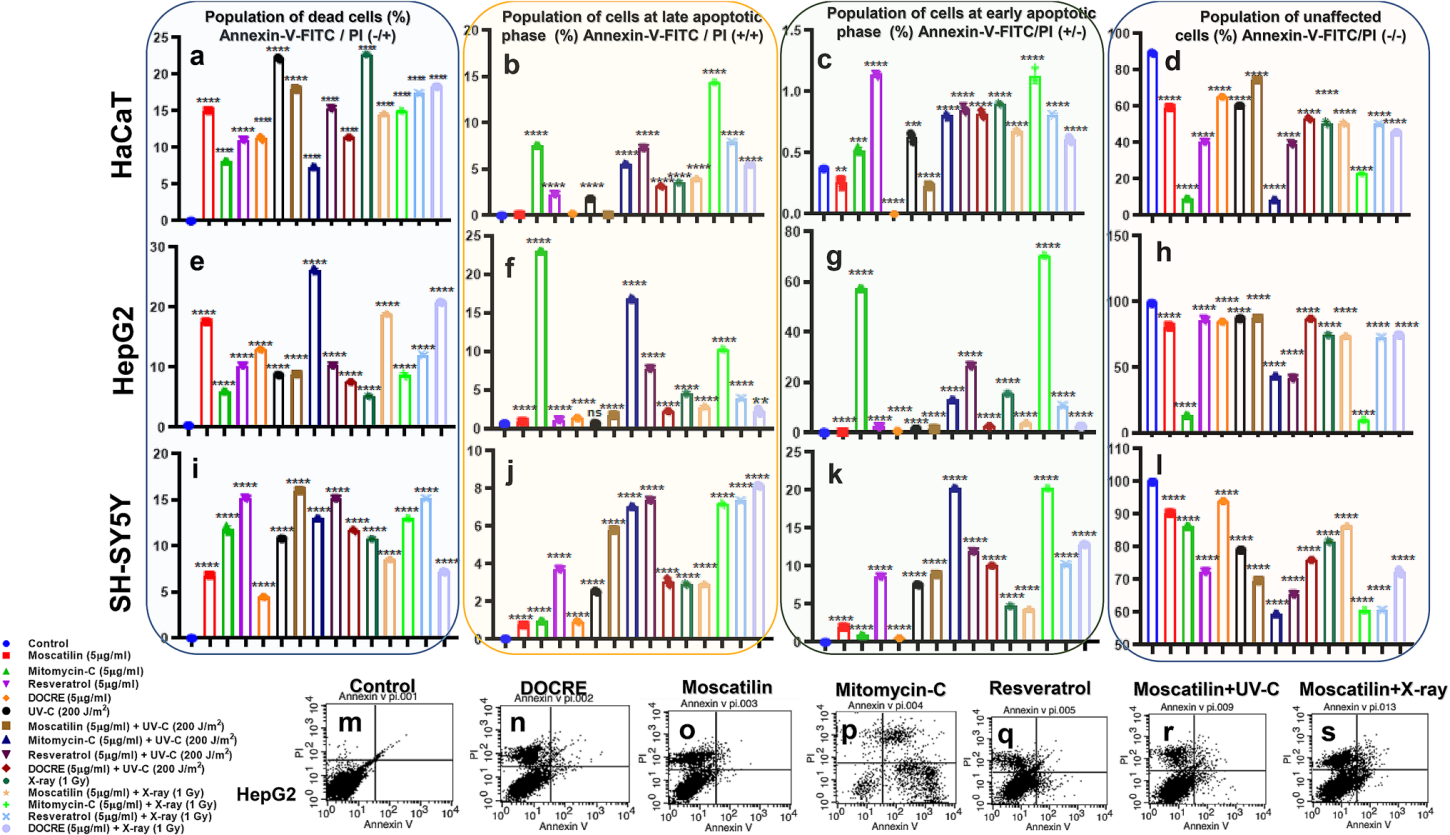
Detection of cell death mode, displaying the cell populations [values represented as percentages] in three studied cell lines, viz., HaCaT, HepG2 and SH-SY5Y under various treatments of X-ray and UV-C radiation along with Moscatilin, Resveratrol, and *Dendrobium ovatum* crude extract (DOCRE) [alone and in combinations]. **a**–**d** Graphical representation displaying sub-populations of HaCaT cells in dead (necrotic) phase, late apoptotic phase, early apoptotic phase, and unaffected cells after various treatments. **e**–**h** Graphical representation displaying sub-populations of HepG2 cells in dead (necrotic) phase, late apoptotic phase, early apoptotic phase, and unaffected cells after various treatments. **i**–**l** Graphical representation displaying sub-populations of SH-SY5Y cells in dead (necrotic) phase, late apoptotic phase, early apoptotic phase, and unaffected cells after various treatments. CellQuest Pro software generated dot plots showing HepG2 cells distributed in 4 different quadrants in **m** control, **n** treated with 5 µg/ml DOCRE, **o** treated with 5 µg/ml Moscatilin, **p** treated with 5 µg/ml Mitomycin-C, **q** treated with 5 µg/ml Resveratrol, **r** treated with 5 µg/ml Moscatilin along with 200 J/m^2^ UV-C, and **s** treated with 5 µg/ml Moscatilin along with 1 Gy X-ray. Data presented as Mean ± SD, with ****P < 0.0001, ***P < 0.001, **P < 0.01, and *P < 0.05 vs control group. All the treatments groups are color-coded

The mode of cell death with Moscatilin was via necrosis with associated apoptosis. However, the apoptotic cell population enhanced when applied along with both UV-C (Fig. 8r) and X-ray (Fig. 8s) radiations in HepG2 cells. DOCRE gave more of necrotic type of cell death (Fig. 8n), when compared to the control (Fig. 8m). The combination with radiation pushed the cells more towards apoptosis. Resveratrol and Moscatilin were almost on par with Mitomycin-C, when the drugs were treated along with X-ray and UV-C. Mitomycin-C alone and in combination with radiations was found to be much more toxic when compared to the other drugs with radiations, and it showed more cells at early and late apoptosis (Fig. 8p) in HepG2 cells. The necrotic cell population was very high in all the treatments (Fig. 8n–s) except for Mitomycin-C.

To improve the cytotoxic activity and antiproliferative efficacy of Resveratrol, scientists have attempted to create structural analogs of the compound by adding methyl groups, modifying either the olefinic bridge or aromatic ring structure, which has improved the activity of the compound (Xin et al. 2020). The improved cytotoxicity of Moscatilin over Resveratrol as observed in the present study is due to the inherent nature of rotatory bridge connecting the benzyl rings and the presence of methyl groups. Since Moscatilin has shown inhibitory effects on HaCaT cell line, it holds the potential to be tested for psoriasis and other skin pathologies.

**Fig. 9:**
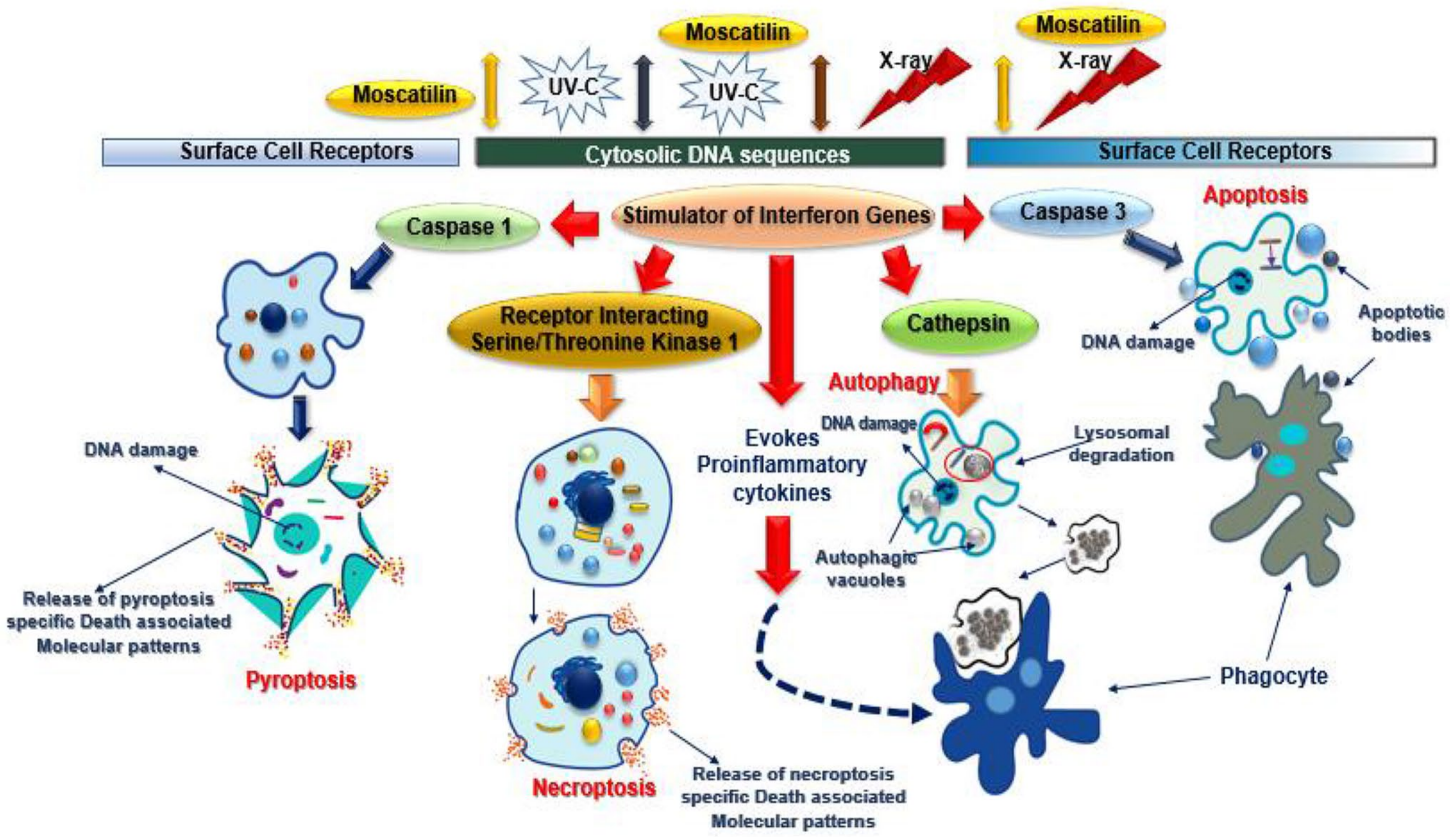
Moscatilin, in unison with radiation, caused immunogenic death of cancer cells starting from Pyroptosis, terminating in Necroptosis. Moscatilin alone could evoke immunogenic cell death. Analyses of DAMPs released would ascertain the mode of cell death caused by Moscatilin

The use of Moscatilin, Resveratrol, and DOCRE in association with radiations has stimulated overlapping death pathways, which could be either necroptosis or pyroptosis associated with autophagy as the population of cells in early apoptosis, and late apoptosis was found to be relatively low. The mode of cell death induced by Moscatilin could be due to pyroptosis culminating in necroptosis (Fig. 9). The death pathways have led to the release of immunogenic molecules that instigated the anticancer effect via immune responses. It is essential to study the function of cell death effectors and intracellular release factors induced by Moscatilin to have a better understanding of the mechanisms and physiological effects caused in association with radiations. Immunogenicity-deprived cancer cells usually avoid destruction via an immune response. Genetic mutation rates are quite low in such cancers and lack the power of expressing de novo antigens. They endorse a tumor-suppressive internal milieu by spawning anti-inflammatory cytokinins. Some chemotherapies possibly can attenuate the immune system, thereby inducing an immunologically silent apoptotic cell death. Release of Damage-Associated Molecular Patterns (DAMPS) and intracellular organelles are hallmarks of necrosis which induce a pathophysiological immune response culminating in necroinflammation (Asadzadeh et al. 2020).

Nearly 70% of the cancer patients undergo ionizing radiation treatment either stand-alone or, in conjugation with the chemotherapeutic or immunotherapeutic regimen. Immunogenic cell death has overpowered cytotoxicity-based cell death. It is widely known that immunogenic cell death is fostered by radiation therapy (Frank and Vince 2019). Radiation induces a blend of signals that stimulate antigen-specific adaptive immune responses. There are shreds of evidence that both chemotherapy and radiotherapy deploy immunogenic cell death pathways (Deng et al. 2020). The current study is an excellent platform to gauge and validate the ability of Moscatilin, Resveratrol, and DOCRE in inducing immunogenic cell death and their efficacy enhancement in combination with radiation. It would be interesting to analyze the type of DAMPs released after specific treatments which would aid in distinguishing the mode of cell death.

## Conclusions

The present study, for the first time, demonstrated the comparative cytotoxic and radiosensitive effect including the induction of apoptosis/necrosis with cell-cycle arrest caused by Moscatilin, Resveratrol, and *D. ovatum* crude extract obtained from the seedlings generated in vitro. The study indicated that, unlike the generic drug Mitomycin-C, Moscatilin did not induce cytotoxicity in healthy cells and could specifically act on cancer cells effectively at a low concentration. Moscatilin functioned as a potential radiosensitizer, resulting in radiation-induced death of cancer cells in the studied cell lines. The morphological features of the cells suggested that Moscatilin alone could evoke immunogenic cell death which is Necroptosis, and in combination with irradiation, it caused Pyroptosis in cells that culminated in Necroptosis.

## Supplementary Information

Below is the link to the electronic supplementary material.

Supplementary file1 (DOCX 258 KB)

## Abbreviations

DOCRE: *Dendrobium ovatum* Crude extract
IC_50_: Half maximal inhibitory concentration
MTT: 3-(4,5-Dimethylthiazol-2-yl)-2,5,diphenyltetrazolium bromide
PE: Plating efficiency
RCF: Relative centrifugal force
SF: Surviving fraction

## References

[CR1] Alara RO, Abdurahman NH, Ukaegbu CI (2021). Extraction of phenolic compounds: a review. Curr Res Food Sci.

[CR2] Asadzadeh Z, Safarzadeh E, Safaei S, Baradaran A, Mohammadi A, Hajiasgharzadeh K, Derakhshani A, Argentiero A, Silvestris N, Baradaran B (2020). Current approaches for combination therapy of cancer: the role of immunogenic cell death. Cancers (Basel).

[CR3] Cardile V, Avola RE, Graziano AC, Russo A (2020). Moscatilin, a bibenzyl derivative from the orchid *Dendrobium loddigesii*, induces apoptosis in melanoma cells. Chem Biol Interact.

[CR4] Cardoso JC, Zanello CA, Chen JT (2020). An overview of orchid protocorm-like bodies: mass propagation, biotechnology, molecular aspects, and breeding. Int J Mol Sci.

[CR5] Chen TH, Pan SL, Guh JH, Liao CH, Huang DY, Chen CC, Teng CM (2008). Moscatilin induces apoptosis in human colorectal cancer cells: a crucial role of c-Jun NH_2_-terminal protein kinase activation caused by tubilin depolymerization and DNA damage. Clin Cancer Res.

[CR6] Chen CA, Chen CC, Shen CC, Chang HH, Chen YJ (2013). Moscatilin induces apoptosis and mitotic catastrophe in human esophageal cancer cells. J Med Food.

[CR7] Chen WK, Chen CA, Chi CW, Li LH, Lin CP, Shieh HR, Hsu ML, Ko CC, Hwang JJ, Chen YJ (2019). Moscatilin inhibits growth of human esophageal cancer xenograft and sensitizes cancer cells to radiotherapy. J Clin Med.

[CR8] Deng H, Zhou Z, Yang W, Lin LS, Wang S, Niu G, Song J, Chen X (2020). Endoplasmic reticulum targeting to amplify immunogenic cell death for cancer immunotherapy. Nano Lett.

[CR9] Frank D, Vince JE (2019). Pyroptosis versus necroptosis: similarities, differences, and crosstalk. Cell Death Differ.

[CR10] Fujimoto M, Bo T, Yamamoto K, Yasui H, Yamamori T, Inanami O (2020). Radiation-induced abnormal centrosome amplification and mitotic catastrophe in human cervical tumor HeLa cells and murine mammary tumor EMT6 cells. J Clin Biochem Nutr.

[CR11] Guan L, Zhou J, Lin Q, Zhu H, Liu W, Liu B, Zhang Y, Zhang J, Gao J, Feng F, Qu W (2019). Design, synthesis and antitumour and anti-angiogenesis evaluation of 22 moscatilin derivatives. Bioorg Med Chem.

[CR12] Hazra B, Ghosh S, Kumar A, Pandey BN (2011). The prospective role of plant products in radiotherapy of cancer: a current overview. Front Pharmacol.

[CR13] Ho CK, Chen CC (2003). Moscatilin from the Orchid *Dendrobrium loddigesii* is a potential anticancer agent. Cancer Invest.

[CR14] Kamaladhasan N, Mohan Raj R, Soundararajan N, IndharSaidanyan R, Saravanan S, Chandrasekaran S, Khasim S, Hegde S, González-Arnao M, Thammasiri K (2020). Beauty of orchid flowers are not adequate to lure indian biologists. Orchid biology. Recent trends & challenges.

[CR15] Kimura H, Lee C, Hayashi K, Yamauchi K, Yamamoto N, Tsuchiya H, Tomita K, Bouvet M, Hoffman RM (2010). UV light killing efficacy of fluorescent protein-expressing cancer cells in vitro and in vivo. J Cell Biochem.

[CR16] Kuruba V, Gollapalli P (2018). Natural radioprotectors and their impact on cancer drug discovery. Radiat Oncol J.

[CR17] Majumder PL, Sen RC (1987). Moscatilin, a bibenzyl derivative from the ocrhid *Dendrobium moscatum*. Phytochemistry.

[CR18] Mohan G, AyishaHamna TP, Jijo AJ, Saradha Devi KM, Narayanasamy A, Vellingiri B (2019). Recent advances in radiotherapy and its associated side effects in cancer-a review. J Basic Appl Zool.

[CR19] Mortezaee K, Najafi M, Farhood B, Ahmadi A, Shabeeb D, Musa AE (2020). Resveratrol as an adjuvant for normal tissues protection and tumor sensitization. Curr Cancer Drug Targets.

[CR20] Müller M, Wang Y, Squillante MR, Held KD, Anderson RR, Purschke M (2018). UV scintillating particles as radiosensitizer enhance cell killing after X-ray excitation. Radiother Oncol.

[CR21] Murashige T, Skoog F (1962). A revised medium for rapid growth and bio assays with tobacco tissue cultures. Physiol Plant.

[CR22] Pai HC, Chang LH, Peng CY, Chang YL, Chen CC, Shen CC, Teng CM, Pan SL (2013). Moscatilin inhibits migration and metastasis of human breast cancer MDA-MB-231 cells through inhibition of Akt and Twist signaling pathway. J Mol Med (Berl).

[CR23] Sarsaiya S, Jain A, Jia Q, Fan X, Shu F, Chen Z, Zhou Q, Shi J, Chen J (2020). Molecular identification of endophytic fungi and their pathogenicity evaluation against *Dendrobium nobile* and *Dendrobium officinale*. Int J Mol Sci.

[CR24] Teixeira da Silva JA, Ng TB (2017). The medicinal and pharmaceutical importance of *Dendrobium* species. Appl Microbiol Biotechnol.

[CR25] Teixeira da Silva JA, Cardoso JC, Dobránszki J, Zeng S (2015). *Dendrobium* micropropagation: a review. Plant Cell Rep.

[CR26] Thomas A, Pujari I, Shetty V, Joshi MB, Rai PS, Satyamoorthy K, Babu VS (2017). *Dendrobium* protoplast co-culture promotes phytochemical assemblage in vitro. Protoplasma.

[CR27] Tiwari P, Mishra KP (2019). Flavonoids sensitize tumor cells to radiation: molecular mechanisms and relevance to cancer radiotherapy. Int J Radiat Biol.

[CR28] Wang YP, Ai J, Luo Y, Li QQ, Li L (2020). The complete chloroplast genome of *Dendrobium wattii* (Orchidaceae). Mitochondrial DNA B Resour.

[CR29] Xin ZH, Meng YL, Jiang WJ, Li YP, Ge LP, Zhang CH, Liu LN, Kang YF (2020). Finding an efficient tetramethylated hydroxydiethylene of resveratrol analogue for potential anticancer agent. BMC Chem.

