# Supplementary material for: Cytotoxicity and radiosensitizing potency of Moscatilin in cancer cells at low radiation doses of X-ray and UV-C
Source: 3 Biotech. 2021 May 20;11(6):281. doi: 10.1007/s13205-021-02827-3 (PMC8137750; doi:10.1007/s13205-021-02827-3)
Supplement: Supplementary file 1 — Supplementary file1 (DOCX 258 KB) [file 13205_2021_2827_MOESM1_ESM.docx]

**Blinded Supplementary Information File**

**Cytotoxicity and radiosensitizing potency of Moscatilin in cancer cells at low radiation doses of X-ray and UV-C**

**Reagents and materials**

*Dendrobium ovatum* capsules (fruits) (Fig. 1b) were collected from the Kudremukh region (Western Ghat zone of Karnataka), India. The plant identity was authenticated by a renowned taxonomist Dr. K Gopalakrishna Bhat, and the voucher specimens were deposited at the herbarium of Manipal School of Life Sciences (MSLS), Manipal, Karnataka, India. Standard solution of Moscatilin (≥95% purity) was purchased from Chengdu Biopurify Phytochemicals Ltd. Sichuan, China. RP-HPLC was performed with Waters Alliance e2695 separations module, including Waters 2487 dual wavelength (ƛ) absorbance UV detector, equipped with Agilent ZORBAX Eclipse Plus C18 column (4.6 mm × 150 mm × 5 µm), Sigma-Aldrich, USA. The screening of *D. ovatum* was performed using Agilent LC/Q-TOF system equipped with an Agilent 1200 liquid chromatography (LC) system consisting of an Agilent 6520 quadrupole time-of-flight mass spectrometer (Q-TOF). The cell lines for cell proliferation assay were obtained from National Centre for Cell Science (NCCS), Pune, India and American Type Culture Collection (ATCC), Virginia, USA, and were maintained in mammalian cell culture laboratory, MSLS, Manipal, Karnataka, India. The human breast carcinoma (MCF-7), human hepatoma (HepG2), human cervical cancer (SiHa), human colorectal cancer (HT-29) and human bone osteosarcoma (Saos-2) were the cell lines purchased from NCCS. Whereas, the human tongue squamous cell carcinoma (Cal-27) and human fibroblasts (the non-cancerous skin tissue cell line as control) used for the assay were purchased from ATCC. Dulbecco’s Modified Eagle Medium (DMEM) supplemented with 4 mg/l folate was purchased from HiMedia Laboratories Pvt. Ltd., Mumbai, India. Fetal bovine serum was bought from Invitrogen, Bangalore, India. HepG2, SH-SY5Y (neuroblastoma cell line from ATCC) and HaCaT (spontaneously transformed aneuploid immortal adult human keratinocyte cell line from NCCS) were used for the clonogenic assay. Apoptosis detection was performed using the FITC Annexin V Apoptosis Detection kit I (Becton Dickinson, BD Biosciences, USA).

**Plant material and growth conditions *in vitro***

The dried parts of the whole plant were collected from the trees and were pulverized using a mixer grinder to estimate and compare the Moscatilin content. The capsules were surface sterilized with 10% Tween-20 detergent for 10 minutes and were washed under running tap water for 30 minutes. The capsules were eventually treated with 0.1% mercuric chloride for 10 minutes. Then the capsules were washed, cut longitudinally, and the seeds were dusted onto half-strength Murashige & Skoog medium (with MS Macroelements-4.23 g/l), supplemented with 1 mg/l Zeatin, 2% sucrose, and the pH was adjusted to 5.8. The medium was solidified with 0.8% Agar. All the medium ingredients for plant tissue culture were purchased from HiMedia Laboratories Pvt. Ltd. Mumbai, India. The culture bottles were incubated in a sterile condition at a temperature of 25 ± 2°C with 12-hour photoperiod maintained by fluorescent lights (Philips, India) with an intensity of 50 µmol m^-2^ s^-1^. Life-cycle of *D. ovatum in vitro* was staged and identified under a stereo zoom microscope (‘Motic’) equipped with the software “Motic images plus 2.0”, and the images related to all the developmental stages were stereomicrographed.

**The ultrasonication low-temperature based extraction procedure**

10 gm of fresh plant tissue was processed on the 75^th^ day. The tissue was macerated to a fine pulp with liquid nitrogen using a mortar and pestle. 100 ml of 100% methanol was added to the pulp. The mixture was pulse-sonicated at 40W for 30 minutes. Throughout the procedure, the samples were kept in ice. The mixture obtained was then centrifuged at 4500RCF for 30 minutes at 4°C. The clear supernatant was concentrated using freeze-drying method till a fine off-white residue was obtained. Samples obtained through the above three extraction procedures were subjected to Thin-Layer Chromatography (TLC), Reversed-Phase High-Performance Liquid Chromatography (RP-HPLC) along with Mass Spectrometry (MS).

**Extraction Procedures**

***The hot extraction procedure***

10 gm of fresh plant tissue was processed on the 75^th^ day. The tissue was chopped and macerated to a fine pulp using a mixer grinder. 100 ml of 70% methanol was added to the ground pulp. The mixture was refluxed for 48 hours using a Soxhlet apparatus. The extract was collected at the 48^th^ hour and was centrifuged at 4500 RCF for 30minutes at 4°C (Eppendorf 5810 R, Hamburg, Germany) to remove suspended particles. The clear supernatant was then freeze-dried till a brown residue was obtained.

***The cold extraction procedure***

10 gm of fresh plant tissue was processed on the 75^th^ day. The tissue was macerated to a fine pulp using liquid nitrogen. 100 ml of 70% methanol was added to the pulp. The mixture was transferred to a separating funnel and incubated at 4°C. The extract was collected at the 48^th^ hour and was then centrifuged at 4500RCFfor 30 minutes at 4°C to remove suspended particles. The clear supernatant was then freeze-dried till a brown residue was obtained. Replicates of cold extraction were pooled and finally, 7.5 gm powder was obtained.

***The ultrasonication low-temperature based extraction procedure***

10 gm of fresh plant tissue was processed on the 75^th^ day. The tissue was macerated to a fine pulp with liquid nitrogen using a mortar and pestle. 100 ml of 100% methanol was added to the pulp. The mixture was pulse-sonicated at 40W for 30 minutes. Throughout the procedure, the samples were kept in ice. The mixture obtained was then centrifuged at 4500 RCF for 30 minutes at 4°C. The clear supernatant was concentrated using freeze-drying method till a fine off-white residue was obtained. Pooling of 10 replicates gave 8 gm of lyophilised powder. The ultrasonication method gave the highest retrieval of moscatilin and hence was used for the preparation of DOCRE from in vitro samples.

***Confirmation of Moscatilin from dried in situ and in vitro samples of Dendrobium ovatum***

***Purification of Moscatilin***

60 gm of dried *in vitro* tissues and *in situ* dried samples were pulverized separately into a fine powder with 100 ml of ice-cold methanol. The sample was pulse-sonicated (Vibra-Cell^TM^, ultrasonic liquid processor, Sonics & Materials Inc, Connecticut, USA) at 40W for 30 minutes and stored in the cold solvent at -20°C for a week. The ultrasonication was carried out 4 times during the storage. The sonicated samples were centrifuged at 5000 RCF for 30 minutes, and the filtrate (~80 ml) was collected. Using a rotary evaporator, the filtrate was concentrated, and the volume was reduced to 50 ml. The concentrated 50 ml filtrate was diluted with Milli-Q water (100 ml) and was re-extracted with 50 ml of diethyl ether using a separation funnel. The diethyl ether layer (upper layer) from the filtrate (35 ml) was fractionated with 2N NaOH (35 ml). Both the upper layer as well as the lower layer of the filtrate were acidified with 2 ml of concentrated HCl and were subjected to Thin-Layer Chromatography (TLC), Reversed-Phase High-Performance Liquid Chromatography (RP-HPLC) along with Mass Spectrometry (MS).

***Moscatilin Identification***

TLC Aluminium oxide 60 F 254, Basic 5 × 20 cm glass plates, Merck, KGaA, Darmstadt, Germany, were used for identification. Various solvent systems were used to purify and resolve closely related compounds. The mixture of hexane: ethyl acetate (7:3) was found to be optimum for better separation. 10 µl of samples were applied at equidistant spots. Once the samples reached 3/4^th^ the distance of the solvent front, the plates were removed from the TLC chamber. The plates were retained in an oven at 65°C for 10 minutes. Chromatograms developed were viewed at 314 nm and 254 nm using a handheld UV lamp. Later 50 µl of samples were equidistantly loaded. Each chromatographed band was scraped out from the TLC plates and analyzed using mass spectrometer for the identification of Moscatilin. Samples from 20 such plates were scraped, centrifuged at 4500 RCF for 15 minutes to pellet down the aluminium oxide by dissolving in methanol. The supernatant was lyophilized, which gave a white crystalline powder and was subjected to tandem mass spectrometry (MS/MS) for confirmation.


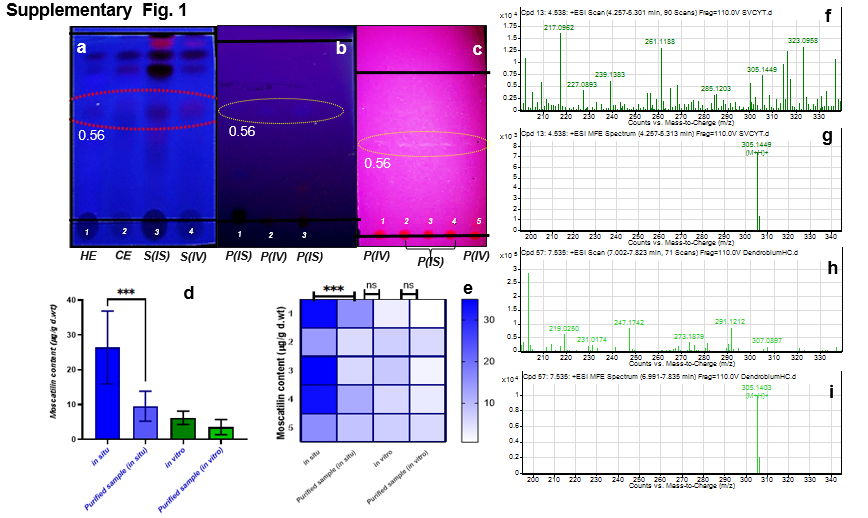


**Supplementary Fig. S1 a.** Targeted MS/MS performed to confirm the presence of active principle, Moscatilin in *Dendrobium ovatum in vitro* samples. **b.** HPLC chromatogram of *Dendrobium ovatum in situ* samples displaying the peak of Moscatilin (in red), prior purification. **c.** HPLC chromatogram of *Dendrobium ovatum in vitro* plantlets displaying the peak of Moscatilin (in red), prior purification. **d.** HPLC Chromatogram showing the peak of Moscatilin standard. **e.** HPLC Chromatogram showing the peak of Moscatilin after TLC-based purification from *Dendrobium ovatum in vitro* plantlets.


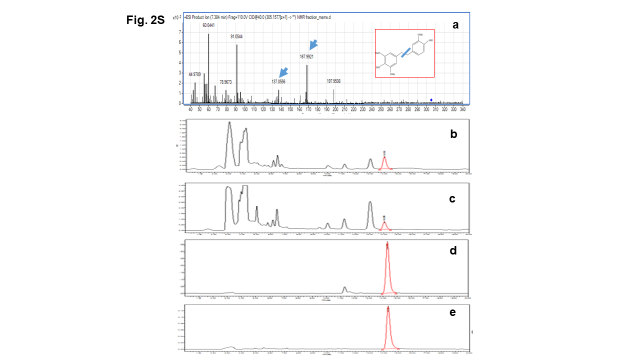


**Fig. S2 a** Targeted MS/MS performed to confirm the presence of active principle, Moscatilin in *Dendrobium ovatum in vitro* samples. **b** HPLC chromatogram of *Dendrobium ovatum in situ* samples displaying the peak of Moscatilin (in red), prior purification. **c** HPLC chromatogram of *Dendrobium ovatum in vitro* plantlets displaying the peak of Moscatilin (in red), prior purification. **d** HPLC Chromatogram is showing the peak of Moscatilin standard. **E** HPLC Chromatogram is showing the peak of Moscatilin after TLC-based purification from *Dendrobium ovatum in vitro* plantlets.

**The treatment groups involved in Clonogenic assay**

The clonogenic assay is used to determine the colony formation after treatment by staining it with crystal violet dye. When the cells attained 80% confluence, the total cell count and viability assessments were performed using trypan blue staining. Based on the cell count and doubling time, 300 cells/ml were seeded onto 6-well plates (test and control) with triplicates. Growing cells were subjected to the following treatments,

**Set 1:** HepG2, SH-SY5Y and HaCaT were independently treated with DOCRE in triplicates. The concentrations of DOCRE used were 1, 10 and 12.5 µg/ml.

**Set 2:** HepG2, SH-SY5Y and HaCaT were separately subjected to X-ray irradiation in triplicates. The radiation was imparted to the cells by the instrument (Faxitron X-ray, 43855F - CP160 Option, Lincolnshire, Illinois, USA). The dosages of X-ray used were 1, 3 and 5 Gy.

**Set 3:** HepG2, SH-SY5Y and HaCaT were individually subjected to UV-C radiation in triplicates. The radiation was imparted to the cells by Stratagene Statalinker 2400 UV Crosslinker. The dosages of UV-C radiation used were 20, 200 and 2000 J/m^2^.

**Set 4:** HepG2, SH-SY5Y and HaCaT were exclusively subjected to 5 µg/ml DOCRE along with the selected dose of X-ray (1 Gy).

**Set 5:** HepG2, SH-SY5Y and HaCaT were discretely subjected to 5 µg/ml DOCRE along with the selected dose of UV-C (200 J/m^2^).

**Set 6:** HepG2, SH-SY5Y and HaCaT were independently treated with Moscatilin in triplicates. The concentrations of Moscatilin used were 1, 10 and 12.5 µg/ml.

**Set 7:** HepG2, SH-SY5Y and HaCaT were separately subjected to 5 µg/ml Moscatilin along with the selected dose of X-ray (1 Gy).

**Set 8:** HepG2, SH-SY5Y and HaCaT were independently subjected to 5 µg/ml Moscatilin along with the selected dose of UV-C (200 J/m^2^).

**Set 9:** HepG2, SH-SY5Y and HaCaT were independently treated with Resveratrol in triplicates. The concentrations of Resveratrol used were 1, 10 and 12.5 µg/ml.

**Set 10:** HepG2, SH-SY5Y and HaCaT were individually subjected to 5 µg/ml Resveratrol with the selected dose of X-ray (1 Gy).

**Set 11:** HepG2, SH-SY5Y and HaCaT were separately subjected to 5 µg/ml Resveratrol with the selected dose of UV-C (200 J/m^2^).

**Set 12:** HepG2, SH-SY5Y and HaCaT were independently treated with Mitomycin-C in triplicates. The concentrations of Mitomycin-C used were 1, 10 and 12.5 µg/ml.

**Set 13:** HepG2, SH-SY5Y and HaCaT were individually subjected to 5 µg/ml Mitomycin-C with the selected dose of X-ray (1 Gy).

**Set 14:** HepG2, SH-SY5Y and HaCaT were separately subjected to 5 µg/ml Mitomycin-C with the selected dose of UV-C (200 J/m^2^).

Thus, every cell line had 14 sets of treatment (individual and in combinations). All treatment series for every cell line had exclusive untreated control plates in triplicates.

Cell survival capacity and cell death were observed by imaging through an inverted phase-contrast microscope. The survival rate was estimated through colony count in the form of plating efficiency (PE) (%) and surviving fraction (SF).

**Plating Efficiency** = (Number of colonies formed)/(Number of cells seeded)×100

**Surviving Fraction** = (Number of colonies formed after treatment)/ (Number of cells seeded)×Plating Efficiency

The percent viability was calculated as follows:

Viability percentage = (Test absorbance-Blank absorbance)/(Control absorbance-Blank absorbance)×100

The colonies were stained with 1 ml of 0.5% crystal violet dye prepared in methanol.
